# The Long-Term Benefits of Positive Self-Presentation via Profile Pictures, Number of Friends and the Initiation of Relationships on Facebook for Adolescents’ Self-Esteem and the Initiation of Offline Relationships

**DOI:** 10.3389/fpsyg.2017.01981

**Published:** 2017-11-15

**Authors:** Anna Metzler, Herbert Scheithauer

**Affiliations:** Developmental Science and Applied Developmental Psychology, Freie Universität Berlin, Berlin, Germany

**Keywords:** adolescents, Facebook use, self-presentation, profile pictures, number of friends, self-esteem, initiation of relationships, computer-mediated communication

## Abstract

Social networking sites are a substantial part of adolescents’ daily lives. By using a longitudinal approach the current study examined the impact of (a) positive self-presentation, (b) number of friends, and (c) the initiation of online relationships on Facebook on adolescents’ self-esteem and their initiation of offline relationships, as well as the mediating role of positive feedback. Questionnaire data were obtained from 217 adolescents (68% girls, mean age 16.7 years) in two waves. Adolescents’ positive self-presentation and number of friends were found to be related to a higher frequency of receiving positive feedback, which in turn was negatively associated with self-esteem. However, the number of Facebook friends had a positive impact on self-esteem, and the initiation of online relationships positively influenced the initiation of offline relationships over time, demonstrating that Facebook may be a training ground for increasing adolescents’ social skills. Implications and suggestions for future research are provided.

## Introduction

According to the theoretical framework that defines “development as action in context,” individual development entails two aspects: (a) development is seen as the outcome of one’s intentional and goal-oriented behaviors that are related to specific contextual opportunities and (b) such behaviors evoke changes in the individual itself (Silbereisen and Eyferth, 1986). Adolescents are therefore considered as active constructors of their own development (Dreher and Oerter, 1986).

In this perspective, investigating the opportunities of adolescents’ self-presentation and initiation of relationships on social networking sites (SNSs) for two components of adolescents’ psychosocial development, including identity (self-esteem) and intimacy (initiation of relationships offline), is meaningful for several reasons.

First, adolescents are very likely to use SNSs such as Facebook, because they have a substantial need to communicate and stay in contact with their friends (Peter et al., 2005) due to the rising interest in and significance of peers during adolescence (Hartup, 1996; Harter, 1998). Second, identity development interacts with the need for self-presentation (Harter, 1998), with Facebook satisfying this need in different ways. SNSs enable adolescents to demonstrate who they are by means of their Facebook profile and to gain positive feedback for doing so (Valkenburg et al., 2006). Positive feedback is especially beneficial when received from one’s peers (Zimmer-Gembeck and Skinner, 2011), which on Facebook is most likely.

However, presenting oneself to one’s peers in a face-to-face context, especially to other peers, may lead to awkward or anxious feelings (Harter, 1999). Presenting oneself and initiating relationship online can help adolescents to overcome these uncomfortable feelings due to two features of computer-mediated communication (CMC) (Walther, 1996): (a) the asynchronicity of communication, including self-presentation, and (b) reduced visual and auditory cues (Walther, 1996; Valkenburg and Peter, 2011). An important consequence of these reduced cues is that adolescents become less concerned about how others perceive them and, therefore feel fewer inhibition in initiating contacts or presenting oneself (Walther, 1996; Valkenburg and Peter, 2009a; Van Ouytsel et al., 2016). In sum, CMC enables young individuals to experience a higher control over their self-presentation and relationship initiation compared to face-to-face interactions.

Finally, today’s adolescents spend a large amount of time on SNSs as they are the first generation of “digital natives” (Prensky, 2001). For example, Tsitsika et al. (2014) examined across six European countries that 40% of the participants (aged 14–17 years) spent two or more hours daily on SNSs.

Given the great relevance of SNSs in adolescences’ daily lives, it is important to examine the consequences of its usage. Accumulating evidence suggests beneficial effects of different aspects of SNS usage on various psychological outcomes, such as increased life satisfaction (Ellison et al., 2007; Valenzuela et al., 2009), self-esteem (Valkenburg et al., 2006; Van Zalk et al., 2014), subjective well-being (Kim and Lee, 2011; Valkenburg et al., 2011), gaining social support (Quinn and Oldmeadow, 2013; Oh et al., 2014; Van Zalk et al., 2014; Frison and Eggermont, 2015), developing a sense of belonging to a friendship group (Quinn and Oldmeadow, 2013; Oh et al., 2014), as well as reduced feelings of loneliness (Burke et al., 2010; Deters and Mehl, 2013).

Our research extends previous studies in investigating the impact of three different aspects of one’s positive Facebook use, namely positive self-presentation, number of friends and initiation of online relationships, on the developmental dimensions of (a) self-esteem and (b) the initiation of offline relationships. First, we investigate the long-term rather than the cross-sectional outcomes of different positive Facebook behaviors. Second, we examine an adolescent instead of a college sample. The investigation of this age group is relevant because certain SNS functions are related to developmental tasks in adolescence (e.g., Reich et al., 2012; Spies Shapiro and Margolin, 2014). Third, we examine the impact of specific positive Facebook behaviors rather than mere usage (e.g., frequency of use). Finally, we take positive feedback in terms of the frequency of receiving Likes from one’s Facebook friends as a mediating variable into account.

By examining the association of the usage of different Facebook features such as self-presentation, number of friends and the initiation of online relationships on adolescents’ self-esteem and their ability to initiate relationships offline we wish to illuminate the psychological process of adolescents’ SNSs use and its potential psychological benefits. We chose these specific aspects of positive Facebook behaviors because they represent crucial behaviors for adolescents’ psychological development in an online environment, such as communication and interaction with one’s friends as well as presenting one’s identity to significant others and getting feedback for doing (Hartup, 1996; Harter, 1998).

### Dimensions of Positive Facebook Behaviors

#### Positive Self-Presentation

“Self-presentation can best be understood as selectively presenting aspects of one’s self to others” (Valkenburg and Peter, 2011, p. 122). It is practiced on Facebook when an individual creates his or her own profile, whereby multiple options for presenting oneself are provided (Zhao et al., 2008; Pempek et al., 2009; Lee et al., 2014).

In our study, we focus on self-presentation through profile pictures because it has been posited as the most important instrument for self-presentation on SNSs (Strano, 2008; Ivcevic and Ambady, 2013; Wu et al., 2015). The profile picture is the main representation of the profile owner (Strano, 2008; Wu et al., 2015). It is the picture that accompanies the name of the profile owner and the first picture that potential new Facebook friends see before they send a friend request. It appears alongside every chat, comment, or “Like” of the user. Adolescents also indicated that they looked at the profile pictures on Facebook to find out more about a potential romantic partner, as this enables them to assess the character and personality of the other user (Van Ouytsel et al., 2016).

**FIGURE 1:**
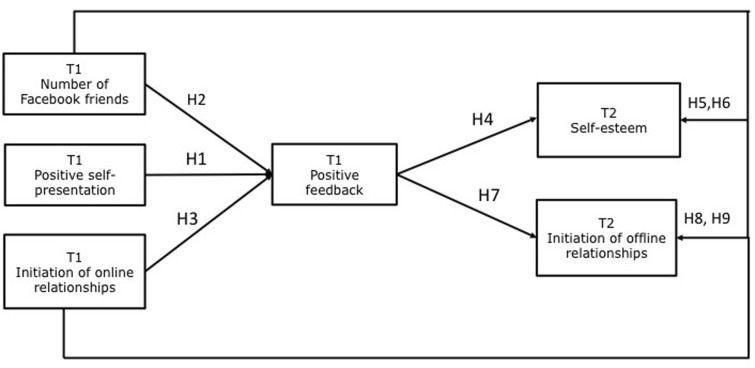
Hypothesized model. For figure clarity, gender is not included in the figure.

As social media users are strategic in self-presentation (van Dijck, 2013), the number of impressions of themselves individuals try to create is almost limitless. Research on strategic self-presentation in face-to-face environments has demonstrated that people are more likely to present themselves in an enhancing manner in an attempt to make the best possible impression (Schlenker and Leary, 1982). Previous findings about online self-presentation demonstrate that in an SNS environment as well users have a tendency to present themselves positively (Strano, 2008; Zhao et al., 2008; Gonzales and Hancock, 2011) as well as authentically (Yang and Brown, 2016). People present themselves positively in the attempt to achieve social goals (Schlenker and Leary, 1982), such as to get others to like them or to convince others of their competences and (social) skills (Jones, 1990). Several studies have associated different aspects of self-presentation among adults on Facebook and positive responses from the SNS audience (Liu and Brown, 2014; Yang and Brown, 2016). Positive self-presentation via profile pictures was found to predominantly elicit positive feedback in terms of receiving Likes on Facebook rather than evoking comments or getting shard (Kim and Yang, 2017). One longitudinal study among adolescents suggests that active public Facebook use (e.g., frequency of posting videos on Facebook) is associated with positive responses on Facebook (Frison and Eggermont, 2015), but the study did not examine specific positive self-presentational behaviors. Therefore, to our knowledge, so far no study links adolescents’ self-presentation to positive feedback. To fill this gap, we hypothesize: Positive self-presentation will increase positive feedback from one’s Facebook friends (**H1**). **Figure 1** summarizes the above-mentioned hypotheses.

#### Number of Friends

“Friends” in the context of Facebook refers to the number of people the user is connected with on Facebook. In contrast to public online chat-rooms in which interactions are primarily based on anonymous communication between unacquainted individuals, SNSs such as Facebook involve non-anonymous communication with known people from one’s offline world (Valkenburg and Peter, 2007). Multiple studies have shown that adolescents’ online and offline worlds are related (Lenhart and Madden, 2007; Pempek et al., 2009; Van Zalk et al., 2014). Facebook friends have basically two functions: one the one hand they provide the audience for one’s self-presentation on Facebook, on the other hand they can actively participate in the users’ behaviors on Facebook by sending private messages, commenting, sharing or liking the actions of the other users. As a consequence, online friends become an important source of emotional and practical support (Boyd, 2006), which in turn may contribute to mental health in adolescence (Frison and Eggermont, 2015). Therefore, the number of friends on Facebook is considered as an important aspect of one’s positive Facebook use.

Several studies support a positive association between the number of friends and different forms of social feedback (Kim and Lee, 2011; Manago et al., 2012; Nabi et al., 2013; Oh et al., 2014), positive interaction (Oh et al., 2014); life satisfaction (Manago et al., 2012; Oh et al., 2014) and well-being (Kim and Lee, 2011; Manago et al., 2012; Nabi et al., 2013). It seems reasonable that a bigger network would increase the likelihood of receiving positive feedback on Facebook. Nevertheless no study so far addressed the association of the number of friends and positive feedback. Therefore, we assume: Number of friends will increase positive feedback from one’s Facebook friends (**H2**).

#### Initiation of Online Relationships

The desire for social belonging is a fundamental motive to initiate and maintain social relationships (Baumeister and Leary, 1995) and at the same time is one of the main motives to create a SNS profile (Ellison et al., 2007; Nadkarni and Hofmann, 2012; Reich et al., 2012; Spies Shapiro and Margolin, 2014).

Peer communication on SNSs is highly desirable because SNSs provide an environment in which the rules of the larger offline world can be practiced and reinforced (Yang and Brown, 2013). The deep connection to others requires affirmation in the virtual environment, so that users perceive that the information they share with others is positively received (Liu and Brown, 2014).

In the present study, we refer to the initiation of online relationships, in earlier studies also labeled the initiation of friendships, as a part of interpersonal competence (e.g., Buhrmester, 1990). Adolescent friendship demands greater facility in a number of close relationship competencies than in childhood, as they must be able to initiate conversations and relationships outside the classroom context (Buhrmester, 1990) such as nowadays for instance in online environments. Youths who are incapable of initiating relationships may fail in developing intimate friendships. A lack of intimate friendships in turn may lead to fewer validating interactions with peers. As a result adolescents feel less secure, more anxious, and less worthy (Buhrmester, 1990).

Adolescents who initiate contacts on SNSs more often may profit from these efforts in two interrelated ways. First, due to adolescents’ more frequent initiation of contacts with peers, other users become more aware of them. Second, they may be perceived as more socially likeable and therefore other users may be more willing to give positive feedback regarding their other Facebook behaviors (e.g., liking their profile pictures). This assumption follows the stimulation hypothesis that online communication leads to closer friendships among adolescents and that SNSs generally stimulate social interaction (e.g., Valkenburg and Peter, 2007).

Previous research among adolescents focuses primarily on the association of online communication and internalizing problems following the compensation hypothesis. It states that young individuals who are uncomfortable interacting with peers in face-to-face contexts are able to meet their social needs through SNSs more easily and therefore benefit from their online communication (McKenna et al., 2002). As certain channels of communication such as eye contact, tone of voice (e.g., shaking, high pitched) and facial expressions are not available on SNSs, introverted (Peter et al., 2005), shy (Orr et al., 2009), lonely (Bonetti et al., 2010; Teppers et al., 2014), and social anxious adolescents (Selfhout et al., 2009; Bonetti et al., 2010; Teppers et al., 2014) seem to profit from online communication. For example, the findings of Bonetti et al. (2010) found that lonely children and adolescents were motivated to use online communication significantly more frequently to compensate for their poorer social skills offline and by doing so fulfilled crucial needs of social interaction, self-disclosure and identity exploration. To expand the focus on the association of internalizing problems and adolescents’ online communication, we hypothesize: Initiation of online relationships will increase positive feedback from one’s Facebook friends (**H3**).

### Two Components of Adolescents’ Psychosocial Development: Self-Esteem and Initiation of Offline Relationships

#### Self-Esteem

All theories on self-esteem agree that individuals have the desire to maintain, protect, and enhance their self-esteem (Rosenberg, 1989). Peer acceptance and interpersonal feedback on the self as well as the control over one’s environment are significant predictors of adolescents’ self-esteem and well-being (Harter, 1999, 2003). SNSs may provide adolescents with all three aspects. As discussed in the previous section, SNSs enable adolescents to control what they want to present to others and to initiate relationships in a safer context compared to face-to-face interactions (Walther, 1996; Valkenburg and Peter, 2009a; Van Ouytsel et al., 2016). This is important since an insufficient self-esteem among adolescents is considered to result in poorer mental and physical health during later life (Trzesniewski et al., 2006). Following Mead’s (1934) theory of symbolic interaction, people internalize and experience themselves indirectly based on the attitudes of others. Therefore, individuals self-esteem reflects the ways others perceive and value them (Heatherton and Wyland, 2003). Feeling accepted by others will increase one’s self-esteem, whereas feeling rejected will decrease one’s self-esteem. In line with this assumption, accumulating evidence among adults suggests a positive association of receiving affirmation on SNSs and self-esteem (Gonzales and Hancock, 2011; Yang and Brown, 2016; Burrow and Rainone, 2017).

Little attention, however, has been paid to the impact of interpersonal feedback online from one’s peers on adolescents’ self-esteem. One early cross-sectional study found that the tone of feedback on their profile was related to individuals’ social self-esteem. Positive feedback was associated with an enhanced self-esteem, and negative feedback with a reduced self-esteem (Valkenburg et al., 2006). Yet the study did not examine the tone of reaction as a result of a specific SNS behavior (i.e., self-presentation) but merely as a result of the frequency of SNS use. By experimenting with their self-presentation, they can optimize the reactions and feedback from their peers and thus enhance their self-esteem (Valkenburg and Peter, 2011). Thus, we hypothesize: Positive feedback from one’s Facebook friends will increase adolescents’ self-esteem (**H4**).

Based on the “Capitalization Theory” (e.g., Sas et al., 2009), the number of friends on Facebook might remind the users of their social connections, which in turn would directly increase their self-esteem. According to the notion of a “friends” heuristic (Nabi et al., 2013), the number of friends might predict self-esteem directly, because Facebook users are considered to apply a heuristic based on their number of friends to evaluate social support availability. Social support availability in turn might be perceived as a source of mental health benefits, such as self-esteem. Thus, we propose: The number of friends will have a direct positive impact on adolescents’ self-esteem (**H5**).

Moreover, initiating social contacts with peers may foster self-esteem by increasing a feeling of social connectedness to others (LaRose et al., 2001). Facebook offers adolescents the opportunity to talk about topics they like, such as music, or to share videos on a common interest, which may facilitate the initiation of relationships online and in turn have a positive impact on their self-esteem. Also online communications may enhance the feeling that one has a satisfactory number of communication partners to interact with (Denissen et al., 2008) since every interaction with other users on Facebook is stored and can be retrieved at any time. Thus, we propose the following hypothesis: Initiation of online relationships will have a direct positive impact on adolescents’ self-esteem (**H6**).

#### Initiation of Offline Relationships

As the initiation and maintenance of friendship networks is considered a developmentally significant process during adolescence (Hartup, 1996), it is crucial to examine how the use of SNSs contributes to “offline” social skills such as the initiation of offline relationships. Analogous to our understanding of the initiation of online relationships, we refer to the initiation of offline relationships as an aspect of interpersonal competence.

As positive feedback received from friends on SNSs was found to be the source of enhanced self-esteem (Valkenburg et al., 2006), it may be plausible that positive responses to one’s positive Facebook behaviors may also contribute to adolescents’ ability to initiate relationships online. Positive feedback may give adolescents the encouraging experience they need to initiate offline relationships confidently. Thus, we hypothesize: Positive feedback from one’s Facebook friends will increase adolescents’ initiation of offline relationships (**H7**).

The network size will also positively influence adolescents’ ability to initiate offline relationships more easily in two interrelated ways. First, a higher number of friends offers the user more opportunities to communicate with a higher number of people offline due to the online-offline connection. Second, as Facebook displays a diverse range of information about every other user on the “newsfeed” section, such as events visited or activities undertaken, adolescents can build on this information to start an offline conversation. Thus, we propose the following hypothesis: The number of friends will have a direct positive impact on adolescents’ initiation of offline relationships (**H8**).

Through the initiation of online relationships, adolescents can practice and reinforce their communication abilities with a large number of other teens. These online communication opportunities in turn may carry over to adolescents’ offline lives, so that their offline social competence will improve (Valkenburg and Peter, 2008). Indeed, adolescents indicate that they use online contexts to strengthen offline relationships (Reich et al., 2012). Instant messaging with peers, which is comparable with sending private messages on Facebook, was for example found to have a positive impact on adolescents’ existing offline friendships (Valkenburg and Peter, 2009b). This positive effect can be explained by adolescents’ tendency to disclose intimate information more frankly online than they might do offline (Valkenburg and Peter, 2009b).

Nevertheless, research on the impact of specific online behaviors on offline social skills is scarce. One study determined that adolescents’ online communication with a wide variety of people stimulated their offline social competence (Valkenburg and Peter, 2008). No study so far has investigated whether practicing social skills within SNSs may carry over to the users’ offline lives. Social interactions on SNSs might be especially beneficial for developing social skills due to the multiple features to interact with others. In addition they are perceived as less threatening than face-to-face interactions due to the heightened control over the interaction (e.g., Valkenburg and Peter, 2011). Thus, we hypothesize: The initiation of online relationships will have a direct positive impact on adolescents’ initiation of offline relationships (**H9**).

### The Hypothesized Model

Taken together, following the assumption of CMC (Walther, 1996) that online communication enables adolescents to experience more control over their self-presentation and interaction with other peers, and the stimulation hypothesis (e.g., Valkenburg and Peter, 2007) that the usage of different SNSs features stimulates young individuals interaction, we formulated the posited hypotheses. In addition, positive feedback could also indirectly increase adolescents’ self-esteem and their initiation of offline relationships over time, as previous research confirms the beneficial role of positive feedback online for individuals’ development (Valkenburg et al., 2006; Manago et al., 2012; Nabi et al., 2013; Yang and Brown, 2016). SNSs are designed to allow users to engage in different supportive interactions such as sharing and providing information, giving somebody encouragement or expressing appreciation, which may produce differential outcomes (Oh et al., 2014). Especially in the period of adolescence positive feedback received from one’s peers is crucial (Zimmer-Gembeck and Skinner, 2011) due to rising interest in and significance of peers (Hartup, 1996; Harter, 1998). For instance, Frison and Eggermont (2015) did not find support for a significant positive impact of active public Facebook use on adolescents’ perceptions of friend support. Only when positive feedback such as positive comments and Likes from the Facebook community were entered in their model, there was a positive relationship between active public Facebook use at T1, positive feedback at T2, and perceived friend support at T2 (Frison and Eggermont, 2015). These results highlight the role of positive feedback in the relation between different types of Facebook use and adolescents’ well-being as a mediating factor. Consequently, we tested the indirect effects between the T1variables positive self-presentation, number of friends, and initiation of online relationships and T2 self-esteem and T2 initiation of offline relationships with the frequency of Likes as a mediating variable.

## Materials and Methods

### Sample and Procedure

The current study pursues a longitudinal approach by using online questionnaire data at two measurement points. Using survey questionnaire is considered appropriate as our approach primarily deals with participants’ individual differences and psychosocial characteristics. In this case survey questionnaires can help ensure the study’s external validity and provide generalizability (Wrench et al., 2013).

At the first measurement point (September 2013 to January 2014), the URL of the online questionnaire was distributed via two channels: on spickmich.de, a German SNS, and in different Facebook groups that deal with the interests of adolescents. 869 participants retrieved the questionnaire. 703 remained after excluding participants who did not match the age range (14–17 years), who did not complete the questionnaire or who answered unreliably by speeding through the questionnaire. At the end of the questionnaire, participants had the opportunity to leave their e-mail address to receive the link for the online questionnaire at the second measurement point. 567 participants left their e-mail addresses to get an invitation for the second part of the study. All participants gave informed consent at both measurement points as approved by the Ethics Board of the FU-Berlin which did not deem parental consent necessary. The participants were also informed that they are allowed to ask for the deletion of their answers at any time as well as that all answers would be treated anonymously.

At T2 (September to November 2014) 295 participants retrieved the questionnaire. 283 participants actually started the survey by entering their identity code; this number was reduced to 241 subjects after excluding participants who did not fit the age range, who had no Facebook profile anymore, or answered the questionnaire double or stopped after giving their identity code. After matching the samples, 217 subjects remained after eliminating participants whose identity code could not be matched or did not complete the questionnaire. The average time span between survey completion in wave 1 and wave 2 amounts to 10 months.

The final sample consists of 148 girls (68.2%) and 69 boys (31.8%). Participants ranged in age from 14 to 18 years (*M* = 16.7; *SD* = 1.03). Most (63.1%) were attending college-preparatory school (Gymnasium), 12.9% were attending vocational school (Realschule), 2.3% general/mixed school (Hauptschule) – all of which are different forms of German secondary schools – 18.4% were attending other forms of school; 3.2% reported not going to school anymore.

To examine whether attrition biased our sample, we examined the differences between those who participated in both waves and those who participated in one wave. More specifically, using Pillai’s trace, a MANOVA showed significant differences, *F*(6,615) = 3.196, *p* = 0.004, $\eta_{p}^{2}$ = 0.03. Follow-up univariate analyses revealed that adolescents who participated in both waves scored significantly lower on positive self-presentation and on initiation of online relationships. Little’s (1988) Missing Completely At Random test indicated that the data were missing completely at random, χ^2^(2) = 3.704, *p* = 0.157.

### Measures

*Number of Facebook friends* was measured by asking how many people were listed as “friends” in a participant’s Facebook profile. Since number of friends is a count variable that has a floor of zero and no ceiling, any distribution drawn from such a population would be expected to be positively skewed and thick-tailed. Indeed, the variable revealed both skewness (4.43_t1_; 6.76_t2_) and kurtosis (34.12_t1_; 70.32_t2_). Because this violates the normality and homoscedasticity assumptions of regression models, we used a log-normalized distribution in our longitudinal model that showed much improved skewness and kurtosis.

*Positive self-presentation* was measured by a 5-item scale that assesses the extent to which participants selectively show positive aspects of themselves through profile pictures on Facebook. Each of the five items had five response categories, ranging from 1 (*never*) to 5 (*very often*). The scale formed a unidimensional structure and showed satisfactory internal consistencies in both waves. A complete list of the of the positive presentation variables is provided in **Table 1**.

**Table 1:** Mean values, standard deviations, and reliabilities of study variables.

	Time 1			Time 2		
		
	Mean	*SD*	ω	Mean	*SD*	ω
**Positive self-presentation**	9.50	3.13	0.68	9.95	3.44	0.63
How often do you use a profile picture that…						
(1) … shows you making a funny or happy mimic or gesture?						
(2) … shows you with friends?						
(3) … shows you striking a pose?						
(4) … shows you with your romantic partner?						
(5) … shows you engaging in your hobby?						
**Number of friends**	290	271.88		298	315.98	
**Initiation of online relationships**	13.09	3.11	0.74	13.13	2.98	0.76
How easy or difficult was it for you in the past 6 month to…						
(1) … to send somebody a friend request?						
(2) … to send a message to someone you don’t know very well?						
(3) … to write on the wall of another user you don’t know very well?						
(4) … to comment on status updates or profile pictures of another user you don’t know very well?						
**Positive feedback**	3.56	0.98		3.62	0.98	
**Self-esteem**	21.77	5.55	0.92	22.19	5.79	0.92
**Initiation of offline relationships**	11.70	3.49	0.83	11.83	3.57	0.85
How easy or difficult was it for you in the past 6 month to…						
(1) … to start a conversation with somebody you don’t know very well?						
(2) … to introduce yourself to somebody you don’t know very well?						
(3) … to start a new friendship with someone you don’t know very well?						
(4) … to give somebody a call you don’t know very well?						

*The reliability of the composite scores was estimated by McDonald’s ω.*

*Positive feedback* was assessed by asking the participants to rate the frequency of Likes that they received in response to their self-presentation through profile pictures on a 5-point Likert scale that ranged from 1 *(never)* to 5 *(always*).

*Self-esteem* was assessed with one subscale of the “Inventar zu Selbstkonzept und Selbstvertrauen” (“Inventory of self-concept and self-confidence”) (Fend et al., 1984), which is an adaption of Rosenberg (1965) self-esteem scale. The subscale contains eight items (e.g. “In general I’m satisfied with myself” or “In my opinion, I’m ok”) using a 4-point Likert scale, ranging from 1 (*disagree*) to 4 (*agree*). Four items were reversed and therefore later converted back.

*Initiation of online and offline relationships on Facebook* were assessed with two subscales, each with four items. The items were adopted from Valkenburg and Peter (2008), based on several earlier instruments measuring different aspects of social competence among adolescents (i.e., Buhrmester et al., 1988). For our research purposes we used four items of the subscale “initiation of offline relationships.” Response options ranged from 1 (*very difficult*) to 5 (*very easy*). Furthermore, we transferred the items to an online context to assess the ability to initiate relationships and interactions on Facebook. This scale also contained four items with a 5-point Likert scale. Both scales formed a unidimensional structure and showed satisfactory internal consistencies in both waves. A complete item list of the initiation of online as well as offline relationships variables is provided in **Table 1**.

Descriptive statistics and reliabilities of the scales at both waves are provided in **Table 1**. The reliability of the composite scores was estimated by McDonald’s ω (McDonald, 1999) as the use of Cronbachs’s α as either a reliability or internal consistency index has been strongly criticized in the psychometric literature because it is based on the assumption of τ-equivalent items, an assumption only rarely met in empirical data (Sijtsma, 2009). The zero-order correlations among the key variables are provided in **Table 2**.

**Table 2:** Zero-order correlations among the key variables at both measurement points.

Measure	1	2	3	4	5	6	7	8	9	10	11	12	13	14
(1) Gender	1													
(2) Age	-0.14^∗^	1												
(3) Positive self-presentation (T1)	-0.04	-0.01	1											
(4) Positive self-presentation (T2)	-0.05	0.11	0.61^∗∗^	1										
(5) Number of friends (T1)	-0.13	0.14^∗^	0.28^∗∗^	0.26^∗∗^	1									
(6) Number of friends (T2)	-0.15^∗^	0.19	0.27^∗∗^	0.28^∗∗^	0.85^∗∗^	1								
(7) Initiation online relationships (T1)	-0.16^∗^	0.05	0.20^∗∗^	0.22^∗∗^	0.24^∗∗^	0.24^∗∗^	1							
(8) Initiation online relationships (T2)	-0.14^∗^	0.01	0.10	0.21^∗∗^	0.22^∗∗^	0.25^∗∗^	0.61^∗∗^	1						
(9) Frequency of Likes (T1)	0.20^∗∗^	-0.02	0.45^∗∗^	0.41^∗∗^	0.33^∗∗^	0.30^∗∗^	0.13	0.19^∗∗^	1					
(10) Frequency of Likes (T2)	0.16^∗^	0.06	0.28^∗∗^	0.40^∗∗^	0.24^∗∗^	0.29^∗∗^	0.16^∗^	0.19^∗^	0.63^∗∗^	1				
(11) Self-esteem (T1)	-0.26^∗∗^	-0.02	0.20^∗∗^	0.14	0.22^∗∗^	0.23^∗∗^	0.25^∗∗^	0.24^∗∗^	0.18^∗∗^	0.16^∗^	1			
(12) Self-esteem (T2)	-0.27^∗∗^	-0.02	0.11	0.02	0.24^∗∗^	0.21^∗∗^	0.15^∗^	0.21^∗∗^	-0.01	0.11	0.78^∗∗^	1		
(13) Initiation of offline relationships (T1)	-0.19^∗∗^	0.06	0.23^∗∗^	0.22^∗∗^	0.28^∗∗^	0.27^∗∗^	0.59^∗∗^	0.49^∗∗^	0.21^∗∗^	0.24^∗∗^	0.35^∗∗^	0.29^∗∗^	1	
(14) Initiation of offline relationships (T2)	-0.17^∗^	-0.01	0.09	0.19^∗∗^	0.27^∗∗^	0.26^∗∗^	0.49^∗∗^	0.62^∗∗^	0.12	0.16^∗^	0.23^∗∗^	0.23^∗∗^	0.61^∗∗^	1

*For the variables number of friends (T1 and T2) natural-log transformed variables are used. ^∗^*p* < 0.05; ^∗∗^*p* < 0.01; ^∗∗∗^*p* < 0.001.*

### Strategy of Analysis

Statistical analyses were conducted using the statistical programs IBM SPSS Statistics 22 (SPSS, 2013) and the Structural Equation Modeling software Mplus 7 (Muthén and Muthén, 1998–2012), using the maximum likelihood method. The final longitudinal sample of 217 students was used. A longitudinal path analysis was conducted using continuous variables, computed through mean scores (except for the number of Facebook friends and positive feedback). The bootstrapping method was used to assess the significance of indirect pathways (MacKinnon, 2008).

In the longitudinal model, T2 self-esteem and initiation of offline relationships were regressed on the T1 mediator positive feedback for profile pictures (H4 and H7), and this mediator was regressed on the T1 variables positive self-presentation, number of Facebook friends, and initiation of online relationships (H1–3). To test the direct effects, T2 self-esteem was regressed on the T1 number of friends (H5) and the T1 initiation of online relationships (H6). Moreover, T2 initiation of offline relationships was regressed on the T1 number of friends (H8) and the T1 initiation of online relationships (H9). Because Pillai’s trace showed that there were gender differences, *F*(10,186) = 3.361, *p* = 0.000, $\eta_{p}^{2}$ = 0.058, in positive feedback for profile pictures at T1 and T2 with girls scoring higher, and in number of Facebook friends at T2, the initiation of online relationships at T2, and the initiation of offline relationships at T1 and T2 with boys scoring higher, we controlled for the baseline values of participants’ gender by adding them as predictors for all of the hypothesized endogenous variables in our model (i.e., positive feedback at T1, self-esteem at T2, and initiation of offline relationships at T2). Participants’ age was not included in the model because the previous multivariate test did not reveal any effects at any measurement point, Pillai’s Trace *F*(30,564) = 1.168, *p* = 0.249, $\eta_{p}^{2}$ = 0.058. We further allowed control paths between T1 self-esteem, T1 initiation of offline relationships and T1 positive feedback, following the assumptions that individuals with a higher self-esteem and a higher ability to initiate interactions offline might have more chances to elicit positive feedback as they probably have healthier relationships. Furthermore, we added prior values as control variables. More specifically, self-esteem at T1 predicted self-esteem at T2 and initiation of offline relationships at T1 predicted the initiation of offline relationships at T2.

## Results

**FIGURE 2:**
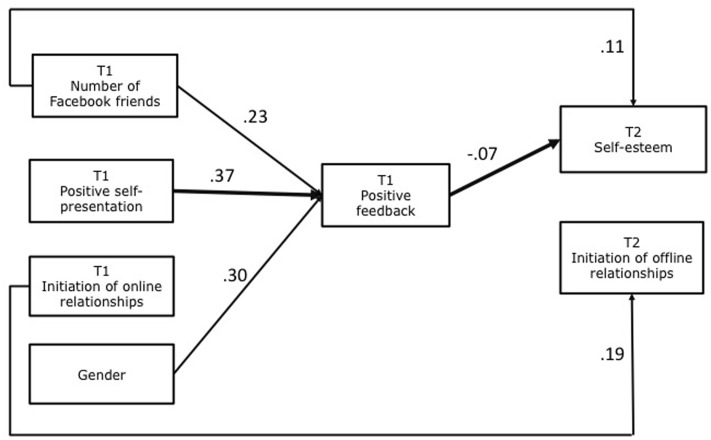
Model examining the relationship between aspects of one’s positive Facebook use, and gender and the outcomes self-esteem and initiation of offline relationships. The reported statistics are standardized coefficients. All displayed paths are significant. The thin lines represent direct paths. The thick lines represent an indirect path.

The model fit the data well on all of the conventional goodness-of-fit indices: χ^2^(2) = 5.290, *p* = 0.36, RMSEA = 0.039, CFI = 0.997, TLI = 0.983, SRMR = 0.01. Path coefficients of the model are presented in **Table 3**. **Figure 2** visualizes the observed path model. The coefficients in **Figure 2** are standardized betas. In line with H1 and H2, T1 positive self-presentation and number of friends were associated with positive feedback at T1 (β = 0.37, *p* = 0.000; β = 0.23, *p* = 0.001). However, T1 initiation of online relationships was not related to positive feedback at T1 (H3) (β = -0.03, *p* = 0.722). Contrary to expectation, positive feedback was not related to T2 initiation of offline relationships (H7) (β = 0.03, *p* = 0.544). Also contrary to our expectations was the only significant indirect path: T1 positive self-presentation was negatively related to T2 self-esteem via T1 positive feedback (H4) (β = -0.07, *p* = 0.001). As expected, T1 number of friends was related to a higher level of T2 self-esteem (H5) (β = 0.11, *p* = 0.034) and T1 initiation of online relationships was positively related to T2 initiation of offline relationships (H9) (β = 0.19, *p* = 0.003). But T1 number of friends was not related to T2 initiation of offline relationships (H8) (β = 0.11, *p* = 0.109), and there was no association between initiation of online relationships at T1 and T2 self-esteem (H6) (β = -0.06, *p* = 0.295). Females reported a more frequent positive feedback at T1 (β = 0.30, *p* = 0.000).

**Table 3:** Path analysis results.

	*b*	*SE*	β (95% CI)
**Direct paths of interest**			
T1 positive self-presentation → T1 positive feedback	0.12***	0.02	0.37 (0.27, 0.47)
T1 number of friends → T1 positive feedback	0.64**	0.19	0.23 (0.10, 0.36)
T1 initiation of online relationships → T1 positive feedback	-0.01	0.03	-0.03 (-0.19, 0.13)
T1 positive feedback → T2 self-esteem	-1.07***	0.29	-0.18 (-0.27, -0.09)
T1 positive feedback → T2 initiation of offline relationships	-0.12	0.20	0.03 (-0.14, 0.08)
T1 number of friends → T2 self-esteem	1.95*	0.92	0.11 (0.01, 0.22)
T1 number of friends → T2 initiation of offline relationships	1.16	0.73	0.11 (-0.03, 0.25)
T1 initiation of online relationships → T2 self-esteem	0.01	0.03	-0.06 (-0.16, 0.05)
T1 initiation of online relationships → T2 initiation of offline relationships	0.21**	0.07	0.19 (0.06, 0.31)
**Controlled paths**			
T1 self-esteem → T1 positive feedback	0.02	0.01	0.11 (0.19, 0.41)
T1 initiation of offline relationships → T1 positive feedback	0.03	0.02	0.09 (-0.06, 0.25)
T1 Gender → T1 positive feedback	0.63***	0.12	0.30 (0.19, 0.49)
T1 self-esteem → T2 self-esteem	0.08**	0.05	0.79 (0.72, 0.86)
T1 Gender → T2 self-esteem	-0.59	0.59	-0.05 (-0.14, 0.04)
T1 initiation of offline relationships → T2 initiation of offline relationships	0.48***	0.07	0.47 (0.35, 0.59)
T1 Gender → T2 initiation of offline relationships	-0.39	0.43	-0.05 (-0.16, 0.06)
**Indirect paths**			
T1 self-presentation → T1 positive feedback → T2 self-esteem	-0.13**	0.04	-0.07 (-0.11, -0.03)
T1 self-presentation → T1 positive feedback → T2 initiation of offline relationships	-0.01	0.02	-0.01 (-0.05, 0.03)
T1 number of friends → T1 positive feedback → T2 self-esteem	-0.07	0.28	-0.04 (-0.07, -0.01)
T1 number of friends → T1 positive feedback → T2 initiation of offline relationships	-0.08	0.14	-0.01 (-0.04, 0.02)
T1 initiation of online relationships → T1 positive feedback → T2 self-esteem	0.01	0.03	0.01 (-0.02, 0.03)
T1 initiation of online relationships → T1 positive feedback → T2 initiation of offline relationships	0.00	0.01	0.00 (-0.01, 0.01)

*^∗^*p* < 0.05; ^∗∗^*P* < 0.01; ^∗∗∗^*P* < 0.001.*

## Discussion

As “adolescence is traditionally considered to be the period in life when peer influences are most intense” (Kandel, 1986, p. 204), the current study aimed to gain more insight into the impact of different positive Facebook behaviors on adolescents’ self-esteem and the initiation of offline relationships.

In accordance with H1, positive self-presentation at T1 predicted a higher level of T1 positive feedback from the Facebook community. These findings are in line with previous research into the association of positive self-presentation and positive feedback (Yang and Brown, 2016). On the one hand this might be simply explained by the fact that by editing one’s Facebook profile more often, the user more often becomes the focus of attention of the audience. On the other hand positivity expressed through specific profile pictures may pay off. This is even more significant in the light of the findings of Forest and Wood (2012) that people who post status updates expressing high negativity are less liked than people with status updates expressing high positivity. Even a recent neurological research study could demonstrate a positive association between self-presentation via profile pictures and positive feedback on Facebook. Adolescents who viewed photographs posted to Facebook that had received more Likes demonstrated greater activation of neural regions involved in reward processing (Sherman et al., 2016).

As expected, the number of friends was positively related to positive feedback from one’s Facebook friends (H2). Contrary to our expectation, however, there was no relationship between the initiation of online relationships and the frequency of Likes (H3), presumably because of the more private nature of this specific Facebook behavior, which is not visible to the whole Facebook audience. Moreover, the initiation of online relationships might evoke other forms of feedback such as private messages or comments rather than Likes. In line with this assumption, a recent study demonstrates that individuals are more likely to like a post containing photos while they were more prone to comment on posts containing text information (Kim and Yang, 2017).

As recent research demonstrates that “getting feedback on content you have posted” is a major reason for using Facebook for some individuals (Smith, 2014), the questions arises what meaning a Like has for an individual. Since a Like only takes one click, it may be an easy way to express respect, affirmation or support. Although we did not examine whether Likes are directly perceived as beneficial, we believe that a Like clearly expresses a positive appreciative reaction and is generally perceived as such. This assumption builds on several findings regarding the meaning of Likes and their link to other psychological outcomes. Lee et al. (2014) for example found that a Like is positively related to building social capital and to bonding, which expresses the social value of a Like. Scissors et al. (2016) argue that Likes are social cues that are perceived as signals for social appropriateness or social acceptance and therefore may express psychological support and empathy in an online setting. Based on survey data as well as data from content analysis, individuals indicated that Likes represent signals of like-mindedness or support. The fact that adolescents are especially vulnerable to feedback from others (Zimmer-Gembeck and Skinner, 2011) and that positive feedback on Facebook is mostly provided by one’s peers emphasizes the importance of this kind of social valuing.

In contrast to our expectations, T1 positive feedback did not have an impact on T2 initiation of offline relationships. Moreover, we were surprised to find one indirect effect with a relationship that contrasted our expectation: T1 positive self-presentation was negatively related to T2 self-esteem via T1 positive feedback. While some researchers found an association between positive feedback and enhanced self-esteem (Valkenburg et al., 2006; Yang and Brown, 2016) using cross-sectional data, Yang and Brown’s (2016) longitudinal approach did not reveal this relationship. This may be due to the fact that our two measurement points were chosen too close to one another to demonstrate an impact of this kind of lightweight feedback on individuals’ self-esteem and initiation of offline relationships. It is also possible that self-esteem is more sensitive to concurrent stimuli (Yang and Brown, 2016).

Nevertheless, the finding of a negative indirect effect may hold for several reasons. As Likes are an element of one’s self-presentation online, people with certain personality traits may view and value Likes differently than others (Scissors et al., 2016). For instance, people with low self-esteem or socially anxious individuals may value Likes more because they have a greater need to feel socially accepted (Leary et al., 1995). Relying on affirmation from others to feel good about oneself may be an expression of limited self-esteem, which can undermine well-being over time (Kernis et al., 2000).

Besides internalizing problems, there are other personality traits that may have affected our results. For instance, according to Seidman (2013) neuroticism was positively associated with the tendency to express ideal and hidden aspects of the self. Some adolescents in our study may have expressed their ideal rather than their actual self. In this case positive feedback from one’s peers on their presented profile pictures would not be linked to the self-esteem development or would affect it negatively. As we did not assess personality traits in our study, we cannot rule out these variables could modulate our hypothesized relationships.

Additionally, social comparison may cause our finding. Young individuals are particularly likely to engage in social (upward or downward) comparison and these types of comparisons can have a strong impact on their self-esteem (Krayer et al., 2008). SNSs such as Facebook make it easy for adolescents to compare themselves to peers simply by looking through the profile page of another user. For example, female adolescents on SNSs reported a more negative body image after looking at pictures of other females with a high physical attractiveness versus a low physical attractiveness (Haferkamp and Kramer, 2011). Participants in our study may be confronted with especially attractive peers and therefore the positive feedback may not be sufficient enough to boost their self-esteem, or even diminish it. Also, as adolescents present themselves positively on SNSs, so do their Facebook friends. The online exposure to their friends’ positive, socially desirable moments (e.g., visiting a party) may lead to a higher level of social comparison (Neira and Barber, 2014). Similar to the “friendship paradox” (the belief that your friends have more friends than you do) (Forest and Wood, 2012), Scissors et al. (2016) describe a “Like paradox,” whereby people feel that their Facebook friends receive more Likes than they do because their friends have more Facebook contacts to provide those Likes. Individuals with lower levels of self-esteem and higher levels of self-monitoring are more likely to think that Likes are meaningful and consequently feel upset when they do not receive an appropriate number of Likes (Scissors et al., 2016).

Finally, more elaborate feedback (e.g., comments or sharing content) from one’s friends may be perceived as more beneficial than Likes alone and therefore may have affected the psychological outcomes in a longitudinal setting differently. According to Calero (2013) having one’s post shared weighs approximately as much as receiving two comments, each of which has roughly the weight of seven Likes.

While profile pictures are the most important instruments for self-presentation on SNSs (Strano, 2008; Ivcevic and Ambady, 2013; Wu et al., 2015), there are many additional ways to create an online identity (e.g., Pempek et al., 2009; Lee et al., 2014) and benefit from the positive feedback from one’s peers. For instance, Pempek et al. (2009) found that emerging adults used information about religion, political ideology, their work, education, and their preferences for music on their Facebook profiles to express their identity online.

In accordance with H5, T1 number of Facebook friends was positively related to adolescents’ T2 self-esteem. This finding suggests that online friends can be an important source of adolescents’ self-esteem and it is consistent with previous findings that emphasize the beneficial role of number of friends in online settings (e.g., Nabi et al., 2013).

T1 initiation of online relationships, however, did not predict T2 self-esteem (H6). This finding is in line with findings by Van Zalk et al. (2011) among university students. They found that chatting with friends as well as with peers that the user knows exclusively online was not significantly associated with self-esteem longitudinally. They only found for less extraverted individuals that chatting with peers found exclusively online was significantly related to higher self-esteem, as well as to fewer depressive symptoms through heightened supportiveness. Their results confirm the social compensation rather than the stimulation hypothesis (e.g., Peter et al., 2005).

As expected, the initiation of online relationships had a direct positive impact on the initiation of offline relationships (H9). The skills practiced online seem to carry over to an offline context. As the ability to create and maintain new relationships becomes especially crucial in adolescence (Hartup, 1996), this is a very encouraging result. Aside from the theoretical contribution, this finding points to the role of SNSs as a training ground where the initiation of relationships can be trained probably due to the fact that online communication is perceived as less threatening than face-to-face interactions (Valkenburg and Peter, 2007). Therefore, practitioners among adolescent disciplines could be advised to encourage adolescents to use SNSs for communication purposes.

Unlike H8, T1 number of friends was not related to T2 initiation of offline relationships. As argued above, actively making contact seems more important than the mere number of friends.

Finally, gender was found to be a significant predictor, with girls receiving more Likes for their positive self-presentation on Facebook. This finding is in line with previous research that female users receive more reactions and replies on their online blog entries than boys do (Mazur and Kozarian, 2010; Hong et al., 2017). As girls generally are more interested in the social aspect of SNSs (Lenhart and Madden, 2007; Valkenburg and Peter, 2007; Valkenburg et al., 2011), they might try more carefully to make a positive impression with the consequence of getting more positive feedback.

As adolescence is a time of struggle to find a balance between autonomy and connectedness as well as to explore one’s identity (Spies Shapiro and Margolin, 2014), SNSs such as Facebook provide a crucial context for reflecting upon and trying out new identities, for learning and attempting new social skills, and for establishing affiliations. Except for the negative indirect path, our results suggest on the whole positive consequences of adolescents’ Facebook use and thereby confirming the stimulation hypothesis. According to this hypothesis, SNSs enable adolescents in general to self-disclose and present oneself to others more freely in comparison to face-to-face communication (McKenna and Bargh, 2000). Online environments are less threatening contexts in which young individuals can share information about oneself more freely due to reduced visual and auditory cues, and the asynchronicity of communication (CMC, e.g., Walther, 1996). With self-presentation facilitating relationship intimacy, this hypothesis also states that online communication leads to higher social skills among young individuals (McKenna and Bargh, 2000). Adolescents’ connectedness to their peers (number of friends) on Facebook is positively related to receiving positive feedback on the one hand, and to a higher self-esteem over time on the other hand. Friendships in the period of adolescence require trust, self-disclosure (including self-presentation), loyalty and support (Collins and Steinberg, 2006) and Facebook opens multiple options for these behaviors. Through their self-presentation via profile pictures adolescents are able to express their belonging to other peers as well as other positive aspects of oneself. Their initiation of online relationships seems to carry over to their offline social skills. In sum, and in line with previous research, the motives to engage in SNS activities are quite similar to those in face-to-face contexts: to maintain existing offline friendships (Subrahmanyam et al., 2008; Reich et al., 2012), make plans with friends, and get to know people better (Lenhart and Madden, 2007; Pempek et al., 2009). Building on our findings adolescents should be encouraged to engage in these forms of active Facebook activities.

This perspective is in line with the most recent research on the impact of adolescents’ Facebook use on the six “Cs” (competence, confidence, connection, character, compassion/caring, and contribution) of the positive youth development (PYD) framework (Lee and Horsley, 2017). They found that the participants of their study could use Facebook as an effective tool to plan and organize leisure activities which in turn influenced adolescents’ social competence and social connections positively over time (Lee and Horsley, 2017).

Besides multiple benefits of adolescents’ SNSs usage, we are aware that several potential costs of SNS usage emerge, including the risk of becoming a victim of online attacks such as sexual solicitation, the potential for a disproportionate amount of negative feedback, and the possibility of unhealthy social comparisons, as noted earlier. The findings of Dredge et al. (2014) for example suggest that specific self-presentation behaviors in adolescence such as the type of relationship status and the number of friends were related to a higher level of cyberbullying victimization. As adolescence is a period of physical changes, including sexual development (Valkenburg and Peter, 2011), teenage girls were found to present themselves more seductively in profile pictures than boys did in teen chat-rooms (e.g., wearing only underwear) (Kapidzic and Herring, 2011).

### Limitations

In interpreting findings from this study, several limitations should be kept in mind. First, our model is not an exhaustive one. Although we included different types of Facebook behavior thought to have a positive impact on adolescents’ development, there are other predictors, mediators, and identity outcomes that should be studied in the future. For instance, we focused on positive self-presentation via profile pictures because it has been posited as the most crucial instrument for self-presentation on SNSs (Strano, 2008; Ivcevic and Ambady, 2013; Wu et al., 2015). However, studying self-presentation via status updates might also be an important source for online affirmation and further positive outcomes (Forest and Wood, 2012; Deters and Mehl, 2013).

The second set of limitations concerns the design of the study and how some of these variables were measured. The longitudinal design does not allow us to draw causal inferences regarding the relationships among variables due to the correlational nature of the study. For example self-esteem and initiation of offline relationships could have influenced self-presentation at the first measurement point, especially given the small time span between the two waves. Future studies should thus use experimental settings as much as possible to show causal effects. Besides the question about the meaning of Likes, one might discuss the accuracy of the self-reported frequency of Likes, since previous research has shown that individuals tend to overestimate the rate of feedback on SNSs (Bernstein et al., 2013). Moreover, the use of a 5-point Likert scale to define positive feedback might be too subjective. As noted earlier, individuals with lower levels of self-esteem and higher levels of self-monitoring seem to value Likes more (Scissors et al., 2016) than people with higher levels of self-esteem and lower levels of self-monitoring. As a consequence, a user with low self-esteem/high self-monitoring may be disappointed by getting few Likes per picture whereas a user with high self-esteem/low self-monitoring may be pleased with it. Another limitation concerns the simultaneous acquisition of positive feedback as a mediator variable.

Furthermore, there are additional potential limitations concerning self-report biases. For example, social desirability could cause a response artifact (Edwards, 1957). That applies in the present study especially to questions about the number of friends and the frequency of Likes as these questions may inadvertently draw inflated answers because of adolescents’ desire to appear more popular. Additional measurements, such as judges’ ratings or content analysis, would contribute to clarify this issue.

Thirdly, the high attrition may have influenced the findings. As preliminary analysis have revealed that adolescents who participated in both waves scored significantly lower on positive self-presentation and on initiation of online relationships, it is possible that personality traits or internalizing problems such as shyness or introversion may have affected the results. As previous research shows that shy and introverted individuals have favorable attitudes toward SNSs (Peter et al., 2005; Orr et al., 2009), these users may have been more interested in its usage and therefore in the subject of the study. In the same way, extraverted or self-confident adolescents may have chosen not to participate in both waves, because of a lack of interest. Future studies should therefore investigate potential mediating and moderating variables, such as adolescents’ personality traits, to provide deeper insight into the relationship between positive Facebook use, and adolescents’ psychosocial development. Although the attrition between the two measurement points is an important limitation in our study, high attrition is commonly observed in adolescence samples (e.g., Valkenburg and Peter, 2009b).

The final set of limitations pertains to the sample. We used a non-representative convenience sample. Participants usually have an affinity for the subject addressed by the survey and therefore consider it interesting enough to invest time responding to the questionnaire. Their affinity for the use of Facebook may have influenced the responses and hence the results. Despite the non-representative nature of our study there are some similarities to two representative studies among 1,200 adolescents in Germany in 2013 and 2014 (JIM-study [Youth, Information, and (Multi) Media]; Feierabend et al., 2013, 2014). Both in the JIM-study of 2013 and our study participants at T1 had on average 290 Facebook friends. Also, 78.8% of our participants (T1) visited Facebook 4–7 times a week compared to 75% of participants from the JIM-study of 2013 who visited Facebook daily or several times a week. Further similarities exist between the JIM-study 2014 and our sample at wave 2. Most of the participants were students (78.3% our sample vs. 87% JIM-study 2014) and the majority of them were attending college-preparatory school (63.1% vs. 85% JIM-study 2014). Moreover, the majority of adolescents aged 14–15 (62%) and 16–17 (75%) used Facebook on a daily basis or several times a week compared to 80.4 and 86.6%, respectively, in our sample at T2.

## Conclusion

Despite these limitations, the results from the present study extend prior research by developing an integrated and differential approach to the relationships between specific types of Facebook use, and adolescents’ self-esteem and their initiation of relationships offline. More specifically, by integrating the process of adolescents’ identity and social development into the theoretical framework of CMC (Walther, 1996), the present study found empirical support for the stimulation hypothesis: The initiation of online relationships had a direct positive impact on the initiation of offline relationships, and the number of friends was positively associated with adolescents’ self-esteem over time. SNSs, such as Facebook may serve as a training ground to practice social skills in a less threatening context compared with face-to-face interactions (Valkenburg and Peter, 2007). Besides these positive effects for the psychosocial development of young individuals, we found a negative indirect association of positive self-presentation, the frequency of positive feedback, and adolescents’ self-esteem over time.

## Author Contributions

The present study is based on a personal initiative of the first author AM. The conception, sampling, analysis, and interpretation of the data were executed by AM. HS was providing input, support and feedback for every part of the study. AM drafted the manuscript. Both authors approved the final version of the manuscript. Both researchers agree to be accountable for all aspects of the work.

## Conflict of Interest Statement

The authors declare that the research was conducted in the absence of any commercial or financial relationships that could be construed as a potential conflict of interest.
